# A bioprosthetic ovary created using 3D printed microporous scaffolds restores ovarian function in sterilized mice

**DOI:** 10.1038/ncomms15261

**Published:** 2017-05-16

**Authors:** Monica M. Laronda, Alexandra L. Rutz, Shuo Xiao, Kelly A. Whelan, Francesca E. Duncan, Eric W. Roth, Teresa K. Woodruff, Ramille N. Shah

**Affiliations:** 1 Division of Reproductive Biology in Medicine, Department of Obstetrics and Gynecology, Feinberg School of Medicine, Northwestern University, Chicago, Illinois 60611, USA; 2 Center for Reproductive Science, Northwestern University, Chicago, Illinois 60611, USA; 3 Oncofertility Consortium, Northwestern University, Chicago, Illinois 60611, USA; 4 Simpson Querrey Institute for BioNanotechnology, Northwestern University, Chicago, Illinois 60611, USA; 5 Department of Biomedical Engineering, Northwestern University, Evanston, Illinois 60208, USA; 6 Department of Anatomy and Cell Biology, University of Kansas Medical Center, Kansas City, Kansas 66160, USA; 7 Northwestern University Atomic and Nanoscale Characterization Experimental Center, Northwestern University, Evanston, Illinois 60208, USA; 8 Department of Materials Science and Engineering, Northwestern University, Evanston, Illinois 60208, USA; 9 Department of Surgery, Feinberg School of Medicine, Northwestern University, Chicago, Illinois 60611, USA

## Abstract

Emerging additive manufacturing techniques enable investigation of the effects of pore geometry on cell behavior and function. Here, we 3D print microporous hydrogel scaffolds to test how varying pore geometry, accomplished by manipulating the advancing angle between printed layers, affects the survival of ovarian follicles. 30° and 60° scaffolds provide corners that surround follicles on multiple sides while 90° scaffolds have an open porosity that limits follicle–scaffold interaction. As the amount of scaffold interaction increases, follicle spreading is limited and survival increases. Follicle-seeded scaffolds become highly vascularized and ovarian function is fully restored when implanted in surgically sterilized mice. Moreover, pups are born through natural mating and thrive through maternal lactation. These findings present an *in vivo* functional ovarian implant designed with 3D printing, and indicate that scaffold pore architecture is a critical variable in additively manufactured scaffold design for functional tissue engineering.

## Abstract (web summary)

There is a clinical need to develop a bioengineering system to support ovary transplantation. Here, the authors generate a bioprosthetic ovary using 3D printed scaffolds of varying pore architectures to support follicle survival and ovarian function in sterilized mice.

Patients undergoing treatment regimens that eradicate their disease, such as cancer, may be left with diminished ovary function, including the inability to undergo puberty, early menopause and infertility^1^,^2^,^3^. Current assisted reproductive technologies and hormone restoration procedures, including *in vitro* fertilization and ovarian transplants, do not provide long-term solutions and leave paediatric patients with metastatic disease without options^4^,^5^,^6^,^7^,^8^. Therefore, the oncofertility field is tasked to develop a whole organ replacement that restores long-term hormone function and fertility for all patients. In past work, we and others have sought to create an engineered ovary with biomaterials and isolated follicles^9^,^10^,^11^,^12^,^13^,^14^. Ovarian follicles are spherical, multicellular aggregates that include a centralized oocyte (female gamete) and surrounding support cells, granulosa and theca, that produce hormones in response to stimulation from the pituitary. The spheroid shape of a follicle is critical to its survival in that the support cells must maintain contact with the oocyte until it has matured and is ready for ovulation. Consequently, a three-dimensional (3D) material environment is critical to maintaining these cell–cell interactions and follicle shape^15^,^16^,^17^.

Thus far, there have been several reports of live births from biomaterial implants in mice, and all have used isolated follicles or whole ovarian tissue encapsulated in a plasma clot or similar fibrin hydrogel bead containing growth factor components or purified vascular endothelial growth factor^18^,^19^,^20^,^21^. These results are very encouraging and have validated both the model procedure and the need for graft vascularization for complete restorative organ function of isolated follicles in a biomaterial^18^,^19^. However, hydrogel encapsulation of follicles poses several challenges, especially with respect to the size of anticipated transplants. Specifically, when translating this work to a large animal or human, the implant must house a significantly larger population of follicles and therefore must be considerably larger than those used in mice. At these scales, diffusion limits may become a concern. Future strategies must permit channels within the hydrogels (to facilitate host vasculature infiltration) or including pre-embedded vasculature to sustain follicle viability and circulate follicular hormones. Moreover, the ovary is a heterogeneous organ that compartmentalizes different follicle pools (quiescent and growing) into the cortex and medulla regions that have varying stiffness^5^,^22^. It is believed that this compartmentalization will be critical to providing long-term (multiple decades) function with an implant^23^,^24^. Therefore, a biomaterial strategy that can produce a mimetic construct of spatially varying material properties may be required for optimal implant function and longevity.

3D printing can be used to address all of these future implant requirements^25^,^26^,^27^,^28^,^29^ for creating a human bioprosthetic ovary, a bioengineered functional tissue replacement. As the first steps towards this goal, here, we investigated tortuous 3D printed porous hydrogel scaffolds with murine follicles seeded throughout the full depth of the scaffold layers to create a murine bioprosthetic ovary. We hypothesized that these scaffolds would provide 3D support to follicles; yet, allow for immediate vascularization and ovulation through the designed porosity. 3D printing also provides unprecedented control over scaffold architectural features. It is unclear, however, how manipulation of pore design may influence cell behavior. Only a few studies to date have investigated the influence of pore architecture on cell–matrix interactions and function^30^,^31^,^32^,^33^. Notably, in another study—and the only pore geometry investigation in soft tissue—Freed and co-workers^33^ discovered that a scaffold with a honey-comb structure provided anisotropic mechanical properties that led to improved directional electrical excitation and cell alignment in a cardiac cells *in vitro*. Here, we sought to optimize pore geometry to support murine follicle survival and function in our bioprosthetic ovary. Our promising results validate exploration in future preclinical large animal models.

## Results

### Partially crosslinked gel-phase ink

**Figure 1:**
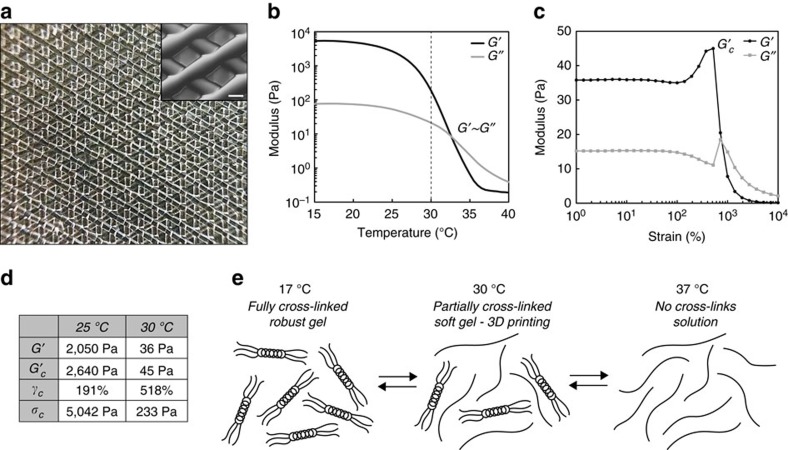
Partially physically crosslinked gelatin achieves high-fidelity printing of scaffolds with ideal tissue engineering properties. (**a**) Photograph of macroscopic view of gelatin 3D printed with 100 μm nozzle, five-layered scaffold. Each layer is discrete and scaffold struts are smooth and continuous leading to a homogeneous pore distribution. Inset: Magnification of scaffold porosity, scale bar 250 μm. (**b**) Gelation profile of 10%. (w/v) gelatin solution. Sol-gel transition occurs at ∼33 °C when G′∼G″. Gelatin was maintained at 30 °C (dashed line) for 3D printing to maintain gelatin in a partially crosslinked state as evident by the fact that the storage modulus of gelatin (30 °C G′ 36 Pa) increased nearly two orders of magnitude upon further cooling (17 °C G′ ∼5,400 Pa). (**c**) Response of 30 °C gelatin to increasing strain. Up to ∼50% strain, the gel exhibited a linear response to strain and after 50% strain, the gel exhibited strain-hardening until the point of catastrophic failure (the critical point), maximum G′ (critical G′ 45 Pa, critical strain 518%, critical stress 233 Pa). (**d**) Summary of rheological properties comparing ideal printable (30 °C) gel to robust, more heavily crosslinked and non-ideal gel (25 °C). Ideal, printable gels have lower G′, critical G′ (G′c), and critical stress (*γ*c) and higher critical stains (*σ*c). (**e**) Schematic of the thermoreversible properties of gelatin. Above 33 °C, gelatin is a solution in which polypeptide chains are separate and soluble. Below 33 °C, local regions of triple helices begin to form and physical crosslink polypeptide chains come together to form the gel. As temperature progressively lowers, more and more crosslinks occur until a fully crosslinked gel forms at ∼17 °C.

A gelatin ink was developed to create a well-defined microporous, bioactive scaffold with extrusion-based 3D printing. Gelatin was selected because it is derived from collagen, an extracellular matrix protein abundant in both human and mouse ovaries^5^,^34^,^35^,^36^, is degradable to allow for cellular remodelling, contains cell adhesion sites and has soft, yet durable mechanical properties^37^. For these reasons, others have reported methods for 3D printing microporous scaffolds of gelatin and gelatin methacrylate^38^,^39^. Prior 3D printing of unmodified gelatin has typically been accomplished by either extruding warm gelatin (solution phase) onto a cold stage to induce rapid gelation^40^,^41^ or directly extruding the cooled, fully crosslinked gel^42^,^43^. The former results in filament spreading and poor layer resolution while the latter results in clumpy, inhomogeneous strands, and thereby irregular pores (Supplementary Fig. 1d). We and others have previously demonstrated the ideal printing properties of gel-phase inks, either partially crosslinked^44^,^45^,^46^ or shear-thinning^47^,^48^,^49^,^50^, over more traditional, solution-phase inks. We, therefore, utilized a partially crosslinked gel state in gelatin by leveraging its thermoresponsive properties^40^. Lowering the temperature of a gelatin solution (<33 °C, Fig. 1b) induces physical crosslinks resulting in regions of triple helix formation (Fig. 1e). We printed gelatin by manipulating the ink temperature to one in-between the liquid (>33 °C) and fully crosslinked gel (<25 °C) state. A lightly crosslinked gel was extruded at 30 °C through a fine diameter nozzle (100 μm) and exhibited smooth and continuous filaments ideal for printing precise porosity (Fig. 1a). When printed onto a cooled stage (10 °C), large, self-supporting objects, the size of human ovaries, were fabricated (2 cm *W* × 5 cm *L* × 0.5 cm *H*=5 cm^3^; Supplementary Fig. 1c). We attribute these ideal printing properties to the fact that the ink was a partially crosslinked gel (*G*′_30°C_ nearly two orders of magnitude less stiff than *G′*_17°C/plateau_) that was weak enough for extrusion yet had solid characteristics necessary to be self-supporting. A more thorough investigation of the rheological properties of this ink revealed that in comparison to non-ideal gels (at 25 °C), the ideal, printable gels (30 °C) were weaker (lower *G′*), yielded at higher strains (critical strain >500%) and had lower critical stresses (Fig. 1c,d and Supplementary Fig. 1b). These differences are consistent with the trends we previously observed between printable and unprintable gel formulations using a partially, chemically crosslinked bioink platform^44^ and suggests that there are common mechanical characteristics that define ideal printability in gel-phase inks. In these studies, we extended the principle of partially crosslinked inks using physically crosslinked (i.e., thermally induced) hydrogels. We used this ink for the creation of well-defined microporous architectures to test the 3D support required by the spherical ovarian follicle.

### Follicle survival is dependent on pore geometry

**Figure 2:**
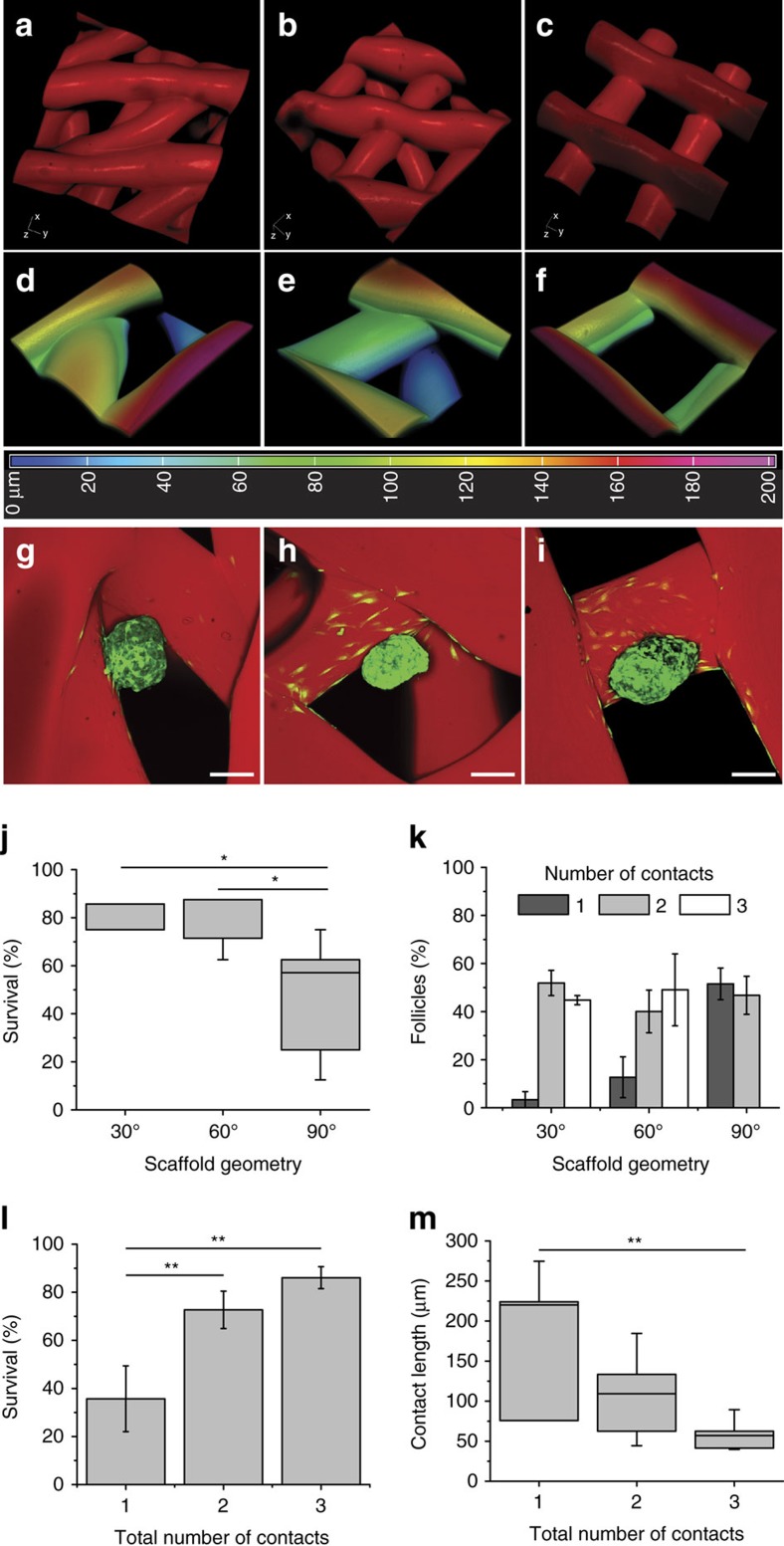
Ovarian follicle survival is dependent on multiple follicle–strut contacts provided by 30° and 60° scaffold pore geometries. (**a**–**c**) 3D reconstructions of confocal fluorescence image stacks of 30° (**a**,**d**,**g**), 60° (**b**,**e**,**h**) and 90° (**c**,**f**,**i**) advancing angle scaffolds. Struts ∼250 μm and distance between struts (from edge to edge) ∼350 μm. (**d**–**f**) 3D reconstructions of confocal fluorescence image stacks of corresponding pores. Colour corresponds to depth of pore according to heat map and total thickness of image is 200 μm. (**g**–**i**) Maximum intensity projections of confocal fluorescence image stacks of GFP+ follicles seeded in pores, after 2 days in culture. Follicles in 30° and 60° pores tended to reside in corners whereas follicles in 90° pores were more likely to be along only one strut (**j**), survival as a function of scaffold geometry. Follicles thrived best in 30° and 60° scaffolds. *P*=0.0146. (**k**) Percentage of follicles making 1, 2 or 3 scaffold contacts per geometry. When seeded into 30° and 60° scaffolds, follicles were most likely to have 2 or 3 scaffold contacts whereas follicles in 90° scaffolds had an equal chance of making only 1 contact versus 2 or more. (**l**) Follicle survival significantly increased with the number of scaffold contacts. *P*=0.0053. (**m**) As the number of strut contacts increased, the length of follicle adhesion along one strut decreased. *P*=0.0029. Scale bars: (**g**–**i**) 100 μm. All data are presented as average±s.e.m. Statistical significance performed using one-way ANOVA with a Holm–Sidak's multiple comparisons test (*P<0.05; **P<0.005).

Three scaffold designs were generated by printing different advancing angles with each layer (30°, 60° and 90°; Fig. 2a–c and Supplementary Fig. 2). Scaffolds were chemically crosslinked (EDC/NHS) for thermal stabilization and increased mechanical properties. These mechanical properties were necessary to create biomaterial implants that are easy to handle during surgery and that closely match native ovarian tissue stiffness^22^ (mean compressive elastic modulus of crosslinked gelatin=16.84 kPa; Supplementary Fig. 3). Pore sizes were selected to accommodate both secondary follicles (150–180 μm in diameter) and aggregates of more immature follicles (<100 μm).

In 3D reconstructions of confocal image stacks of rhodamine-labelled scaffolds, 30° and 60° scaffolds were observed to have extended corners and another strut underlying the pore (blue in heat map; Fig. 2d,e) while 90° scaffolds possessed shallow corners without any underlying struts (Fig. 2f). Importantly, all pore depths analysed were equivalent to the height of a single secondary follicle (∼200 μm) so that follicles contacted multiple layers of the scaffold, rather than a single association site. Confocal imaging revealed that cells around the follicle adhered and spread along scaffold struts around the adhesion sites. Maintenance of the follicle's spherical shape between the 30° or 60° scaffolds and the 90° scaffolds differed during *in vitro* culture (Fig. 2g–i and Supplementary Fig. 5a–c). Follicle survival was scored over an 8-day culture period, which has been previously shown to be a duration required for downstream functional analysis^51^. Generally, follicles spread along scaffold struts in the 90° scaffolds, and by day 8 of culture, many follicles (48.47±8.31%) had died due to dissociation of granulosa cells away from the oocyte. Follicles in 30° and 60° scaffolds supported significantly higher survival rates (78.57±3.57%, 75.89±4.04%, respectively, *P*=0.0146; Fig. 2j). We hypothesized that the increased rate of survival within the 30° and 60° scaffolds was due to more ideal physical support provided by the scaffold struts. To further probe the mechanism of increased survival, the contacts between follicles and struts were counted at 2 days in culture, prior to the increase in observed follicle death that occurred by day 6. Follicles seeded within the 30° and 60° scaffolds had an increased likelihood of making contact with two or more struts (96.67±3.33% and 89.11±6.87%, respectively), while follicles seeded in 90° scaffolds made contact with one strut at a similar rate as making contacts with two struts (51.54±6.56% versus 46.79±7.91%), and did not make contact with three struts (Fig. 2k). We attribute this to the pore geometry of the 90° advancing angle design (short corners and lack of an underlying strut). When examined as a function of number of strut contacts, follicle survival was significantly higher when the follicles made two or three strut contacts over just one strut contact, regardless of the geometry in which they were cultured (one strut, 35.72±13.68%; two struts, 72.70±7.77%; three struts, 86.07±4.531%, *P*=0.0053; Fig. 2l). Follicle survival was positively correlated with the number of strut contacts (Pearson's *r*=0.6966, *P*=0.0027, *R*^2^=0.4852; Supplementary Fig. 4h).

Of additional note, follicle–strut contacts typically spanned a depth of many microns as opposed to just a single point. The longest length of each follicle–strut contact within the XY plane (side contacts) was measured after 2 days in culture for each of the three architectures (Supplementary Fig. 4d–f). The sum of total contact lengths for follicles making contact with one or two struts was not statistically different (198.7±85.58 μm, 164.0±38.47 μm, *P*=0.59; Supplementary Fig. 4g). However, individual side contact lengths significantly decreased with increasing number of total contacts (including a bottom strut, one strut, 198.7±42.78 μm; two struts, 104.4±14.02 μm; three struts, 59.52±5.43 μm, *P*=0.0026; Fig. 2m). These results imply that the follicles cultured *in vitro* required multiple strut contacts to maintain a spherical shape, rather than only one strut contact which resulted in a critical degree of follicle spreading (Supplementary Fig. 5a–c) associated with diminished survival.

**Figure 3:**
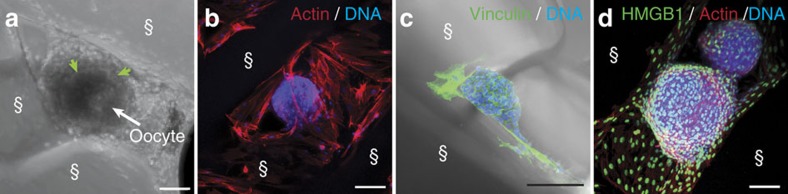
Follicles interact with struts. (**a**) Light microscopy image of follicle cultured within 30° scaffold for 8 days with maintained basement membrane (green arrows) and centralized oocyte (white arrow). (**b**) Maximum intensity projection of confocal fluorescence image stack of follicle stained for actin (red) and DNA (blue) cultured within 60° scaffold for 6 days showing that cells surround and support follicle. (**c**) Maximum intensity projection of confocal fluorescence image stack with light image overlay. Immunostaining of vinculin (green), a cytoskeletal protein associated with cell–matrix adhesions, in the cells that adhere to the gelatin struts of the 3D printed scaffold after 8 days of culture. DNA (blue). (**d**) Maximum intensity projection of confocal fluorescence image stack of follicle cultured for 2 days in 60° scaffold. Immunostaining for HMGB1 (green), counterstained for actin (red) and DNA (blue), indicates that cells surrounding and anchoring the follicle are stromal cells. §, scaffold strut. Scale bars: (**a**) 50 μm; (**b**–**d**) 100 μm.

Additional analysis of follicle–scaffold interactions revealed that murine cells proliferated and spanned the pores in the 30° and 60° scaffolds over the course of the 8-day culture (Supplementary Fig. 5d–g and Fig. 3a). Adhesion of surrounding cells to the scaffold was evident by the presence of actin filaments along the surface of their elongated cell bodies (Fig. 3b) and by cell–matrix focal adhesions as stained by the cytoskeletal protein, vinculin (Fig. 3c). Follicles naturally exist snuggly within the ovarian stroma. However, a reliable marker for these cells did not exist. Using western blot analysis and immunohistochemistry, we discovered and validated that high mobility group box 1 protein (HMGB1) is expressed in the ovary with specific enrichment in stromal cell and oocyte nuclei (Supplementary Fig. 5h–k). HMGB1 is not expressed in theca cells or granulosa cells, and thus distinguishes the somatic stromal compartment of the ovary. Using this marker, we found that the cells that expanded along the struts expressed HMGB1 and thus were likely stromal in origin (Fig. 3d and Supplementary Fig. 5l). In addition, these cells expressed the extracellular matrix protein laminin. Laminin staining is strongly concentrated around the theca cell layer on the periphery of the follicle and is indicative of formation of the follicle basement membrane (Supplementary Fig. 5m,n)^52^.

### Follicles function within 3D printed scaffolds *in vitro*

**Figure 4:**
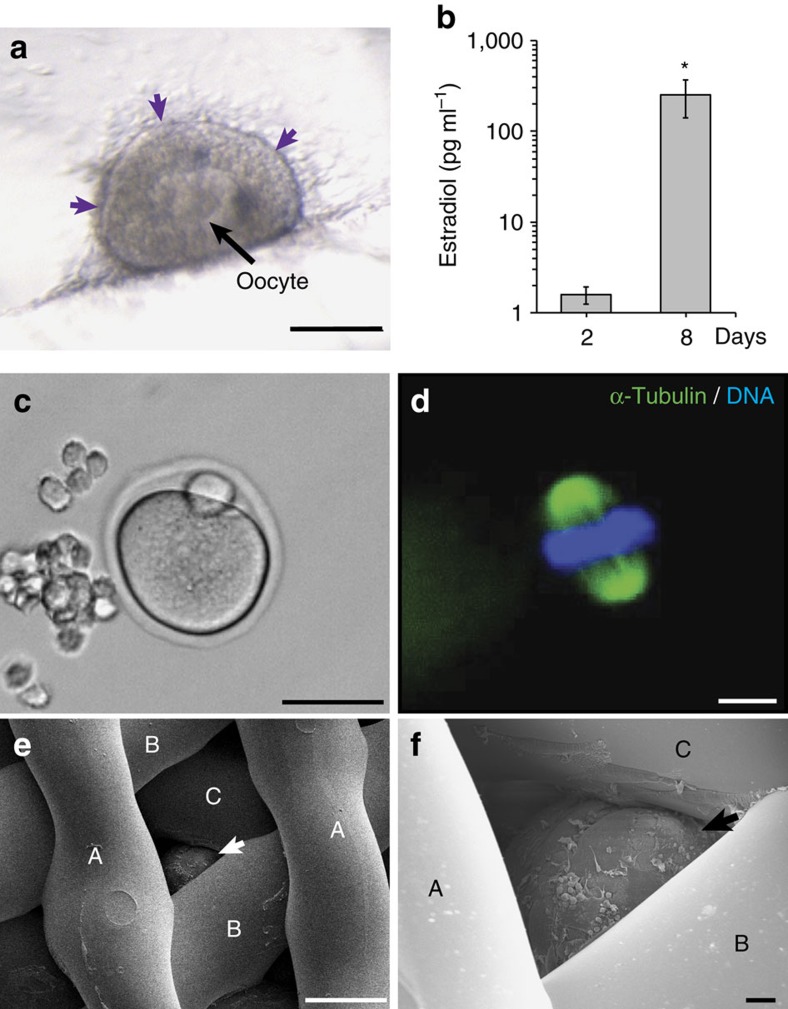
Follicles function within 3D printed scaffolds *in vitro*. (**a**) Light microscopy image of 3βHSD expression (purple) along edge of follicle cultured in 30° scaffold after 4 days of culture. (**b**) Estradiol secretion in media of follicles cultured in 3D printed scaffolds collected at day 2 and day 8 of culture. (**c**,**d**) MII egg with extruded polar body was released from a follicle cultured in 60° scaffold and contains condensed chromatin (blue) along the spindle (green). (**e**,**f**) Scanning electron micrograph demonstrating follicle (arrows) wedged underneath three layers of 60° scaffold struts (identified as layers A, B, C) and cultured for 2 days. Scale bars: (**c**) 50 μm; (**a**,**e**) 100 μm; (**d**,**f**) 10 μm.

Steroidogenesis occurs within the theca and granulosa cells of the ovarian follicles and is the process of converting cholesterol to steroid hormones, such as estradiol and progesterone. These hormones are essential for regulating the reproductive axis (hypothalamus, pituitary, ovary) as well as bone health, breast development and other systemic functions. 3-β Hydroxysteroid dehydrogenase (3βHSD), an essential enzyme in the steroid hormone pathway, was active in the theca cells at the outer layer of follicles cultured in the scaffolds (Fig. 4a), and these follicles secreted an increased amount of the downstream product, estradiol during culture (2 days, 1.26±0.36 pg ml^−1^; 8 days, 255.19±114.21 pg ml^−1^; *P*=0.004; Fig. 4b). We then tested the ability of the scaffolds to support follicles toward terminal differentiation *in vitro* and confirmed that the follicles ovulated fully mature MII eggs with normal polar bodies and spindle morphologies through the scaffold pores (Fig. 4c,d)^53^,^54^. These results indicated that the 30° and 60° scaffold architectures support hormone production, oocyte maturation and ovulation. At this point, we down-selected to the 60° scaffolds for the remaining *in vivo* studies, because of the wider through-pores that allowed for better follicle seeding throughout the entire depth of the scaffold (Fig. 4e,f and Supplementary Fig. 2g,h).

### Bioprosthetic ovary restores organ function in mice

**Figure 5:**
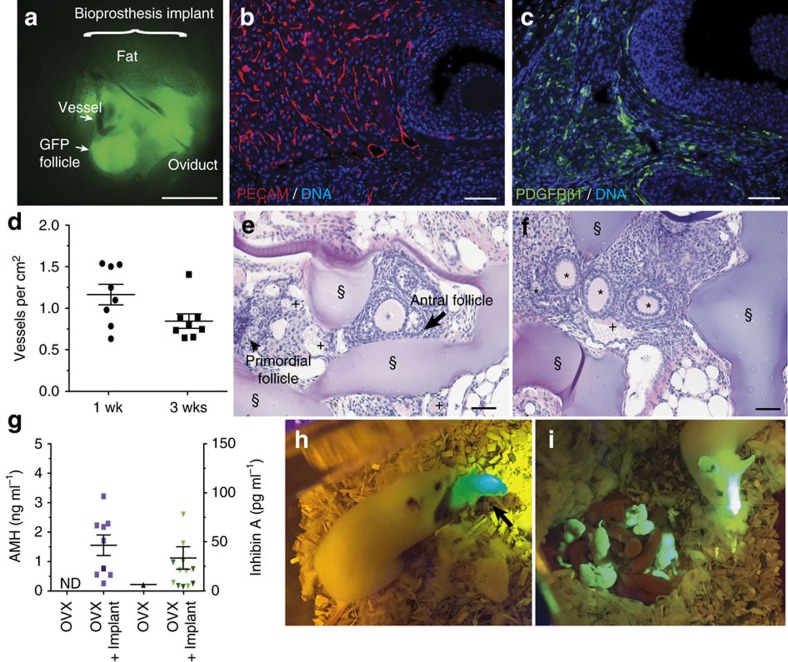
Bioprosthetic ovaries support vascular infiltration and restore function *in vivo*. (**a**) Whole-mount fluorescent image of GFP+ follicles (green) implanted within the ovarian bursa of GFP− mice removed 8 weeks after surgery. (**b**,**c**) Vascularization shown in representative images of immunostaining for endothelial marker platelet endothelial cell adhesion molecule (PECAM) (red) or pericyte marker PDGFRβ1 (green) expression in corpus luteum, antral follicles and interstitial space of bioprosthetic ovary removed 8–10 weeks post-surgery. DNA counterstained blue. (**d**) Quantification of vessels containing red blood cells within ovarian bioprosthesis collected 1 or 3 weeks post-surgery. (**e**,**f**) H&E stained cross-sections of ovarian bioprostheses, removed 3 weeks after surgery, contained vessels and primordial, primary, secondary and antral follicles within the gelatin scaffold struts. (**g**) Serum analysis for peptide hormones anti-Müllerian hormone (AMH) and inhibin A detected within ovariectomized animals with empty scaffold implant (OVX+Sham) or with bioprosthetic ovary (OVX+Implant). (**h**) GFP+ pup (black arrow) born to GFP− bioprosthesis recipient female. (**i**) GFP+ pup born from bioprosthesis sired liters with GFP− CD1 female to create a mixed litter of GFP+ and GFP− pups (grand-pups of ovary recipient). §, scaffold strut. +, vessels. Scale bars: (**a**) 1 mm; (**b**,**c**,**e**,**f**) 50 μm. All data are presented as mean±s.e.m. ND, not detected.

To examine *in vivo* benchmarks of organ function, bioprosthetic ovaries, comprised of ovarian follicles within a 3D printed scaffold that supports follicular function, and sham scaffolds (no follicles) were implanted within the ovarian bursa of adult ovariectomized mice (both ovaries were surgically removed). The bioprosthetic ovaries were constructed by seeding approximately 40–50 small follicles (primordial, primary and small secondary up to 180 μm) isolated from *green fluorescent protein*-positive (*GFP+*) mice in 60° advancing angle scaffolds (2 mm diameter, 1.5 mm thick) and culturing for 4 days *in vitro* (Supplementary Fig. 6a,b) to allow follicles to establish cell–cell and cell–matrix interactions. Spherical *GFP+* follicles were still visible inside the bursa, following removal of the implant at 8 weeks post-surgery, indicating follicle survival within the implant (Fig. 5a and Supplementary Fig. 6e,f). Additionally, the bioprosthetic ovaries became vascularized within 1 week of implantation and without the use of exogenous angiogenic factors (Fig. 5d). Vascularization is essential for circulation of peptide hormones secreted from the follicle. Blood-filled vessels (Supplementary Fig. 6c) were also present at 3 weeks post-surgery and were found alongside follicles representing each stage of folliculogenesis, primordial, primary, secondary and antral follicles, as well as corpus lutea (Fig. 5d–f and Supplementary Fig. 6c,d). Vessels were further confirmed by expression of platelet endothelial cell adhesion molecule, and mural cells were identified throughout the construct by expression of platelet-derived growth factor receptor β1 (Fig. 5b,c). The observed distribution and density of vessels were similar to that observed in normal ovaries^55^.

Orderly folliculogenesis was observed through retention of primordial follicles, and activation of primary, secondary and antral follicles within the bioprosthetic ovary (Fig. 5b,c,e,f and Supplementary Fig. 6c–f). Small and growing follicles secrete peptide hormones, anti-Müllerian hormone (AMH) and inhibin A. Serum analysis of AMH and inhibin A was performed from all surgeries and mice that contained bioprosthetic ovaries had increased levels (OVX+Implant, AMH, 1.51±0.34 ng ml^−1^, inhibin A 32.31±11.33 pg ml^−1^) over ovariectomized sham controls, which contained little or undetectable levels (Fig. 5g). The final step of folliculogenesis is the formation of a corpus luteum (CL) that develops after an egg is ovulated. CLs were present and vascularized throughout the implants (Fig. 5b,c). These data confirm gonadal function at 1, 3 and 8–10 weeks following surgeries.

We then examined the fertility of ovariectomized mice with and without the bioprosthetic ovary. Although both ovaries were removed thereby sterilizing the mice, the reproductive tract, including the oviduct, was left intact to assess fertility through natural mating (Fig. 5a). Two mice with sham controls and seven mice with bioprosthetic ovaries were mated with males who had previously sired pups. Three bioprosthetic ovary recipients had litters of one or two pups each, while none of the sham controls had pups. Of the litters produced, at least one pup per litter was confirmed to have resulted from an egg ovulated from the implant (*GFP+* or black coat; Fig. 5h and Supplementary Fig. 6g, see Methods for scoring). Each of the five pups from these matings were supported by the milk from the lactating, implant-recipient mothers, indicating the mothers received adequate progesterone production from the CL glands to trigger prolactin and produce milk^56^. Once grown to adulthood, the bioprosthesis-derived mice sired (Fig. 5i) or gave birth to their own litters. Hormone restoration and the sequence of fertility events was enabled by the unique, interconnected microporosity of 3D printed scaffolds, which supported vascularization, ovulation and follicle hormone function.

## Discussion

In this work, we investigated how scaffold pore geometry affected the growth and maturation of ovarian murine follicles as well as developed a bioprosthetic ovary that restored ovarian function *in vivo* in mice. Microporous architectures were achieved through 3D printing partially crosslinked, thermally regulated gelatin. We found that specific scaffold architectures created a 3D feel by providing appropriate depth and multiple contact sites for the ovarian follicle, which resulted in optimal murine follicle survival and differentiation *in vitro*. The open micropores within the hydrogel scaffold provided sufficient space and nutrient diffusion for follicle survival and maturation *in vitro* and *in vivo*, as well as space for vasculature to infiltrate when implanted *in vivo* without the need for significant scaffold degradation as is required when using hydrogel encapsulation^22^,^23^. The techniques developed here are the necessary first steps to validate the significant undertaking of exploring such an approach for creating a human bioprosthetic ovary.

Importantly, we accomplish on-platform ovulation through a biomaterial that did not require mechanical manipulation or digestion of the material to release an egg. Mechanical manipulation or enzymatic digestion of the biomaterial may impact the health of the generated egg and therefore reducing intervention by using our scaffolds may be more desirable for future *in vitro* fertility applications such as *in vitro* fertilization. Furthermore, live birth was achieved with the implant alone; angiogenic growth factors, hormone stimulation and embryo transfer were not required^18^,^19^,^51^,^57^,^58^. Since exogenous hormones were not given to the animals, ovulation was triggered endogenously which depends on estradiol and inhibin production from the follicles seeded within the implanted bioprosthesis^59^. Subsequent events also signified that the bioprosthetic ovary was an active participant in the reproductive axis *in vivo*, including ovulation of a healthy egg through the scaffold, and progesterone production from the remaining CL to produce a receptive uterine wall and stimulate lactation. The resulting pups from the bioprosthetic ovary developed normally with their own reproductive competency, as they were all able to sire or deliver healthy litters. These results highlight the high functionality of our bioprosthetic ovary using a scalable and adaptable method.

In future years, the 3D printed bioprosthetic scaffold can be repopulated with ovarian tissues (either native or iPS derived)^60^. The advantage of 3D printing is the opportunity to scale the size of the tissue to the size needed for the transplant recipient (e.g., for a child who is transitioning through puberty or for an adult). Furthermore, the construct could be printed with embedded vasculature to help alleviate nutrient demands in large (multi-cm) tissues. With these first steps presented here, the use of 3D printing will allow for new investigations in reproductive biology. For example, varying stiffness with multi-material printing as well as varying pore size can be used to create a construct that separates quiescent and growing follicle pools, which is necessary for transplant longevity and continued hormone cyclicity^61^,^62^. Future studies will require optimizing the number of cells transferred to the scaffold and the assessment of durable function; these studies are ongoing. While additional experimentation is required to establish that human folliculogenesis will be supported in a similar manner as the mouse follicles shown here, and that the isolated follicles are free of cancerous cells, this bioprosthetic ovary may become a promising solution for restoring hormone and fertility function in oncofertility patients. Outside of reproductive biology, our findings will likely impact others developing tissue units and other spheroid cultures^63^, and underscore the importance of independently investigating the impact of architectural variables when designing scaffolds for other soft tissue and organ targets.

## Methods

### 3D ink preparation and 3D printing

One gram of gelatin (porcine, type A; Sigma-Aldrich) was dissolved in 10 ml of phosphate-buffered saline solution (PBS; Gibco) (pH=7.4) at 37 °C. The solution was subsequently loaded in a stainless steel printing cartridge, and a lab-made agarose (Sigma-Aldrich) piston was placed on top of the solution. The cartridge was then maintained at 30 °C for at least 3 h to cool the solution into a gel. With an EnvisionTEC 3D-Bioplotter, gelatin was printed from a 100 μm stainless steel nozzle onto glass slides maintained at 10 °C. Extruding pressures ranged from 1.8 to 4.5 bar to control ink flow rates, and the ink was printed at a speed of 10 mm s^−1^ into 15 × 15 mm squares, 5 layers thick. The first layer was completely solid (no spacing between struts) whereas the distance between struts (from middle of one strut to middle of adjacent strut) on all subsequent layers was 600 μm. This resulted in scaffolds with struts ∼250 μm and pore size (edge of one strut to the other) ∼350 μm. Three types of microporous internal architectures were printed with advancing angles between layers of 30°, 60° and 90°.

### Scaffold preparation

After printing, structures were kept in a closed container with water (to keep humidity) and on ice. Structures were crosslinked for 1 h with a 15 mM *N*-(3-dimethylaminopropyl)-*N*′-ethylcarbodiimide (EDC; Sigma-Aldrich)/6 mM *N*-hydroxysuccinimide (NHS; Sigma-Aldrich) solution in deionized water to stabilize gelatin scaffolds for culture at physiological temperature. Scaffolds were then washed with deionized water, and sterilized by overnight incubation in 70% ethanol as well as 1 h of UV exposure. Scaffolds were then stored in sterile PBS at 4 °C.

### Ink and scaffold characterization

Oscillatory shear rheology was performed with the gelatin ink on an Anton-Paar MCR 302 rheometer. Rheology was conducted with a cone (2°)-plate fixture and at 10 rad s^−1^. Temperature of the stage was controlled between 15 and 40 °C. Gelatin was loaded onto the stage while warm (37 °C) and after lowering the cone fixture into position, the edges were covered with mineral oil to prevent dehydration. Temperature sweep was conducted at 0.5 °C min^−1^ and at 1% strain. The architecture of scaffolds was analysed from photographs and images were taken with a Photojojo macrolens and cell phone camera along with a Leica M205 C stereoscope. For 3D imaging, scaffolds were fluorescently labelled with NHS-rhodamine (Thermo-Scientific) and were washed with PBS. NHS-rhodamine was dissolved in dimethyl sulfoxide (Sigma-Aldrich) at 10 mg ml^−1^. A diluted PBS solution of the 100 × concentrate was used for labelling for 1–2 h. Labelled scaffolds were then imaged on a Nikon A1R laser scanning confocal microscope. Compression testing was performed on an LF Plus mechanical tester at 0.5 mm s^−1^ (Lloyd instruments, 50N load cell). Gels (200 μl) were prepared between glass coverslips and crosslinked with 15 mM EDC/6 mM NHS solution in deionized water for 1 h to yield flat gel cylinders of 7 mm diameter and 1 mm height. Modulus was taken over 0–10% strain.

### Follicle seeding and culture

Both *in vitro* and *in vivo* experiments designed with scaffolds seeded with follicles are outlined in Table 1. Scaffolds were prepared by using 2, 3 or 4 mm biopsy punches onto the printed design and using a scalpel to lift each piece off the glass slide. A thin microspatula or flat forceps were used to place the scaffold punches on 0.4 μm pore 12 mm transwells (Millipore; PICM01250) in a 24-well plate (Corning; 353047). Each well was filled with 400 μl of growth media (αMEM Glutamax, Life Technologies, 32561) supplemented with 3 mg ml^−1^ bovine serum albumin (BSA) (MP Biomedicals, 210370025), 10 mIU ml^−1^ recombinant follicle-stimulating hormone (from A.F. Parlow, National Hormone and Peptide Program, National Institute of Diabetes and Digestive and Kidney Diseases, Bethesda, MD, USA), 1 mg ml^−1^ bovine fetuin (Millipore, 341506), 5 μg ml^−1^ insulin, 5 μg ml^−1^ transferrin and 5 μg ml^−1^ selenium (Sigma-Aldrich, I1884). Multilayer secondary follicles (150–180 μm) were isolated from 16-day-old CD-1 strain (Harlan) female mice. All mice were housed in a controlled barrier facility at Northwestern University's Center of Comparative Medicine under constant temperature, humidity and light (12 h light/12 h dark). Food and water were provided *ad libitum*. All animal experiments were approved by the Institutional Animal Care and Use Committee and were performed in accordance with National Institutes of Health Guidelines. Ovaries were removed from the bursas and follicles were mechanically isolated with insulin needles in a glass dish containing dissociation media made of Leibovitz's L-15 Medium (Life Technologies, 11415) with 0.5% penicillin–streptomycin (Cellgro, 30-002-CI) and 10% fetal bovine serum (Life Technologies, 10082139). Only follicles that displayed intact morphology were selected for seeding and culture. Follicles were seeded by micropipetting onto scaffolds and removing excess fluid from the bottom of the scaffold. This allowed the follicles to fall through the open porosity and populate the entire depth of the scaffold. Follicles on scaffolds were cultured at 37 °C in 5% CO_2_ in air for up to 8 days. Half of the growth media (200 μl) was replaced every other day. Spent media was stored at −20 °C and analysed for estradiol (below). Follicles were imaged after seeding and at each media change using a dissecting scope (Lecia, MZ95). Follicles were scored as alive if the oocyte was visible, round and generally centralized within the follicle through light microscopy. Four separate experiments with a total of 130 follicles were included and divided into groups of seven or eight follicles. Follicles were excluded if they were not healthy (degenerated oocyte or punctured granulosa cell layer) by 24 h after seeding, as we assumed these follicles were unhealthy because of the isolation procedure. The number of strut contacts was identified by light microscopy on day 2 of culture and scoring was consistent in those that were also observed with confocal microscopy. There were four replicate groups of follicles with one contact, eight groups with two contacts and five groups with three contacts. There were three groups of follicles in 30° scaffolds, six groups in 60° and seven groups in 90° scaffolds. Data in Fig. 2 are represented as the mean±s.e.m. and also listed in the text. An ordinary one-way ANOVA with Holm–Sidak's multiple comparisons test was performed with *α*=0.05. The biological replicates were spread equally among the groups compared and normal distribution was observed among the replicates. The Brown-Forsythe and Bartlett's tests found no significant variation between groups.

### Follicle–scaffold interaction 3D analysis

*GFP*-expressing follicles were seeded into NHS-rhodamine labelled scaffolds and cultured. Two scaffolds per geometry (30°/60°/90°) with four follicles each were analysed (24 follicles total). The experiment was conducted once. After 2 days, the follicles were analysed by confocal fluorescence microscopy (Nikon A1R laser scanning) at an image slice thickness of 5.3 μm. Images were analysed as image stacks as well as 3D reconstructions in NIS Elements software. Confocal images were compared to light microscopy images during culture. Eight follicles were eliminated from analysis because either the follicle moved during transfer for imaging or artifacts in the collected images obscured analysis (e.g., air bubbles between follicle and scaffold). To quantify the number of contacts between follicles and scaffold struts, the image was scrolled through to identify slices where the follicle was flush with the scaffold. Side contacts were measured at the image slice with the longest length of contact (the longest area of green fluorescent follicular cells along a strut). Bottom scaffold contacts were determined if red fluorescence was observed underneath of green fluorescence within 20 μm but were not quantified. Strut contacts underneath the follicle (bottom contacts) could not be quantified due to diminished follicle fluorescence. For individual lengths of contacts per number of contacts, there were four measured lengths for follicles making one strut contact, 11 lengths for follicles making two strut contacts, 12 lengths for follicles making three strut contacts for 17 total follicles. All follicles making three contacts had at least one contact that could not be measured and, therefore, were not included in the total length analysis. For the total length of contact for follicles making one and two total contacts and whose contacts were all side contacts (no bottom, only side contacts were measurable), there four measured total lengths per one and two total contacts for eight follicles total. An ordinary one-way ANOVA with Holm–Sidak's multiple comparisons test was performed with *α*=0.05. The bars in Fig. 2 represent mean±s.e.m.

For scanning electron microscopy analysis, the samples were fixed in a solution containing 2.5% EM Grade glutaraldehyde, 2% paraformaldehyde and 0.1 M PBS for 1 h at room temperature and overnight at 4 °C. Post fixation occurred in 1% osmium tetroxide for 2 h at room temperature followed by 1% uranyl acetate overnight at 4 °C. A graded series of ethanol was used for dehydration before critical point drying in a Tousimis Samdri 795 Critical Point Dryer. The samples were then mounted to scanning electron microscopy stubs with carbon tape and silver paint and sputter coated with 10 nm of AuPd with a Denton Desk IV Sputter Coater prior to imaging. Data were gathered with a Hitachi SU8030 cold field emission scanning electron microscope at 5 kV with working distances ranging from 9 to 20 mm.

### Western blotting

Ovaries were collected from 40-day-old CD1 mice, frozen and stored at −20 °C. Total protein lysate was extracted with a pestle and motor using cell lysis buffer (Cell Signaling Technology, 9803S) in the presence of a protease inhibitor cocktail (Life Technologies, 78440). Denaturing SDS–PAGE was performed with 25 μg of the total protein lysate using standard western blotting techniques on a 4–15% polyacrylamide gel (Bio-Rad, 456–1084). The polyvinylidene fluoride membrane was blocked following transfer using 3% Amersham ECL Prime Blocking Reagent (GE Healthcare, RPN418) and incubated in primary antibodies as follows: 1:1,000 HMGB1 (Abcam, ab18256) and 1:1,000 GAPDH (Cell Signaling Technologies, 5174S) in TBS+0.1% Tween-20. As a control for expression, the HMGB1 blocking peptide (Abcam, ab18650) was also used. A ratio of 1:5 HMGB1 antibody to HMGB1 peptide was incubated overnight at 4 °C prior to application. Membranes were then incubated in horseradish peroxidase conjugated anti-rabbit secondary antibody (GE Healthcare, NA931-1ML), and protein was visualized using Amersham ECL Prime Western Blotting Detection Reagent (GE Healthcare, RPN2232).

### Immunohistochemistry of ovary sections

Ovaries from 40-day-old CD1 mice were fixed in Modified Davidson's Fixative (Electron Microscopy Sciences, 64133-50) and paraffin-embedded. Five micron sections were deparaffinized in Citrisolv (Fisher, 04-355-121) and antigen retrieval achieved using Reveal Decloaker (Biocare Medical, RV1000). Sections were prepared for immunohistochemistry using Avidin/Biotin blocking kit (Vector Labs, SP-2001) in 10% normal goat serum in TBS. Sections were then incubated with diluted 1:100 HMGB1 primary antibody (Abcam, ab18256, lot GR135551-1) in 10% normal goat serum in TBS overnight at 4 °C. As a control for expression, the HMGB1 blocking peptide (Abcam, ab18650) was also used. A ratio of 1:5 HMGB1 antibody to HMGB1 peptide was incubated overnight at 4 °C prior to application. Sections were washed in TBS+0.1% Tween-20 and then incubated with diluted 1:200 biotinylated goat anti-rabbit secondary antibody from the Vectastain Elite ABC kit (Vector Labs, PK-6105) for 2 h at room temperature, washed again and followed by a 30 min incubation in the ABC reagent (Vector Labs, Vectastain Elite ABC kit) and a final wash. Binding was detected with diaminobenzidine (DAB; Vector Labs, SK-4100) for 6 min. Counterstaining was achieved using standard haematoxylin staining. All images were acquired and processed on an Evos fl auto inverted microscope using Evos software (Life Technologies).

### Immunofluorescence of follicles within scaffolds

Constructs were fixed for immunohistochemistry for 30–60 min with 3.8% paraformaldehyde (Electron Microscopy Sciences, 100503-916) with 1% Triton X 100 (Sigma-Aldrich, T8787) and stored in blocking buffer made of PBS containing 0.01% Tween-20 (Sigma-Aldrich, P2287) and 0.3% BSA (MP Biomedicals, 210370025) at 4 °C until ready to use. Samples were washed with PBST. Constructs were blocked and permeabilized with 2% donkey serum, 1% BSA, 0.1% cold fish skin gelatin, 0.1% Triton, 0.05% Tween-20, 0.05% sodium azide in PBS for 1 h. Primary antibodies against vinculin (1:200; Sigma Aldrich, V9131), HMGB1 (1:200; Abcam, ab18256), laminin (1:50; Santa Cruz Biotechnology, sc-20682) were diluted in 10% of the blocking solution and were incubated overnight at 4 °C. After washing with PBST, the samples were labelled with a secondary antibody (1:250 anti-rabbit or 1:1,000 anti-mouse, AlexaFluor 488; Life Technologies A21206, A11008) for 2–6 h, followed by PBST washing and counterstaining with DAPI (1:50; Molecular Probes, D1306) and Phalloidin-555 (actin; 1:50, Abcam, ab176756). 3D constructs were imaged on either a Nikon A1R or C2 laser scanning confocal microscope. Experiments were performed two times with each antibody and a no primary control was also examined for each secondary used.

*In vitro* ovulation was performed on 6–8 days of follicle culture, when the oocytes grew to approximately 70 μm. Follicles were incubated for 16 h at 37 °C in 5% CO_2_ in air in maturation media containing αMEM with 10% fetal bovine serum, 1.5 IU ml^−1^ human chorionic gonadotropin (Sigma-Aldrich, C1063), 10 ng ml^−1^ epidermal growth factor (BD Biosciences, 354010) and 10 mIU ml^−1^ recombinant follicle-stimulating hormone. Released oocytes in metaphase II (MII) were identified and fixed in 3.8% paraformaldehyde containing 0.1% Triton X-100 for 1 h at 37 °C for spindle morphology and chromosome alignment analysis. Oocytes were washed three times in blocking solution with 1 × PBS containing 0.3% BSA and 0.01% Tween-20, incubated overnight in a 1:50 dilution of mouse anti-α-tubulin (Cell Signaling Technology, 5063S) in blocking solution. Then, oocytes were washed three times with blocking solution, mounted using Vectashield containing DAPI (Vector Laboratories, H-1200) and analysed using an EVOS FL AUTO microscope (Life Technologies). Oocytes with barrel-shaped bipolar spindles and well-organized microtubule fibres, along with tightly aligned chromosomes on the metaphase plate, were considered normal^64^.

### 3βHSD staining

To verify the presence of an intact theca cell layer, follicles within the scaffolds were rinsed with PBS then stained with 3βHSD solution containing 0.12 mg ml^−1^ nitroblue tetrazolium chloride (Sigma-Aldrich, N6639), 0.25 mg ml^−1^ β-nicotinamide adenine dinucleotide hydrate (β-NADþ; Sigma-Aldrich, N3014) and 0.025 mg ml^−1^ epiandrosterone (Sigma-Aldrich, E3375) in PBS for up to 3 h at room temperature wrapped in foil^65^. Negative controls were performed on follicles for the same length of time in solution without nicotinamide adenine dinucleotide hydrate β-NADþ and in PBS alone. Cells were considered positive of 3βHSD if they were purplish-brown in colour. Negative controls were not positive for this colour change. This experiment was performed three times with 18 follicles each.

### Hormone ELISAs

Estradiol was detected in the media using an ELISA kit (Calbiotech, ES180S, range of 10–1,000 pg ml^−1^). Individuals who ran the ELISA and interpolated the data were blinded to the treatments (culture day collected). A power analysis determined that three animals per group was sufficient to efficiently detect (80% chance) a minimal difference of 50±10% in serum levels. Terminal blood draws were processed to collect serum, which was tested for AMH and inhibin A by the University of Virginia Center for Research in Reproduction Ligand Assay and Analysis Core. The average of technical replicates was plotted. The reportable range for AMH is 1.56–100.0 ng ml^−1^, and inhibin A is 10–884 pg ml^−1^. Randomized and de-identified serum samples from 5 ovariectomized mice with shams and 16 ovariectomized mice with bioprosthetic ovaries were measured. Zero serum samples from ovariectomized mice with sham and nine with bioprosthetic ovaries were in detectable range for AMH. One mouse with sham and 13 mice with bioprosthetic ovaries had serum containing inhibin A within the detectable range. The bars in Fig. 5g represent mean±s.e.m. Because the mice with bioprosthetic ovaries are expected to have a biological variation of serum levels dependent on the hormone cycle, we did not expect a normal distribution.

### Histological analysis and vessel counting

All tissue processing and haematoxylin and eosin (H&E) staining was performed by the Northwestern University Center for Reproductive Sciences Histology Core. Fixed tissue was processed using an automated tissue processor (Leica) and embedded in paraffin. Serial sections were cut 5 μm thick and selected slides were stained with H&E using a Leica Autostainer XL (Leica Microsystems). Immunohistochemistry was performed on 3–5 sections per sample and at least three samples per group. Each experiment was performed 2–3 times on separate days and included no-primary controls. Sections were imaged on a Nikon E600 Fluorescent microscope (Nikon Instruments) with a Retiga Exi Fast 1394 camera (QImaging). Antibodies against platelet endothelial cell adhesion molecule (1:50; Santa Cruz Biotechnology, sc1506), platelet-derived growth factor receptor β1 (1:100; Abcam, ab32570) or GFP (1:300; Santa Cruz Biotechnology, sc8334) were used and visualized with AlexaFluor secondaries (1:500; Life Technologies, A21206, A21082) or Vectastain Elite ABC Kit (Vector Laboratories, PK-6100). Mounting medium with DAPI counterstain (Vector Laboratories, H-1200) was used to visualize nuclear material.

Every 20th section or ∼100 μm was stained with H&E and imaged on a Nikon E600 microscope. Every vessel containing blood cells within 100 μm of the scaffold was counted for eight ovaries each collected 1 or 3 weeks after surgery. The average area of the scaffold or bioprosthesis boundaries of the first and middle sections were calculated to determine the number of vessels per area of tissue.

### Intrabursal surgeries and mating

Animal use was performed under a Northwestern University Animal Care and Use Committee-approved protocol. Mice ubiquitously expressing the enhanced *green fluorescent protein* (*GFP+*) that resulted from a cross of CD1 strain (white coat, Harlan) and *GFP*-expressing C57BL/6J mice (black coat, Jackson Labs) were used to create the bioprosthetic ovaries and were identified using a BlueStar light with VG1 filter glasses (EMS, BLS-1). Two-millimetre scaffolds were prepared as described above on transwells and with growth medium. Primordial primary and secondary follicles from *GFP+* females were seeded as described above over 2 days, seeding small follicles first and filling to pack entire scaffold on the second day with additional small and some secondary follicles (up to 180 μm in diameter). These scaffolds were cultured for 4 days total *in vitro* prior to surgeries and imaged on the morning before the surgeries. Sham implant scaffolds were prepared in the same way, but did not include cells.

Intrabursal surgeries were performed on 8- to 10-week-old NSG females (white coat; Jackson Labs). Mice were anaesthetized with a mixture of 100 mg kg^−1^ of ketamine and 15 mg kg^−1^ of xylazine. The upper uterine horn, oviduct and ovarian bursa were visualized and brought out of the body cavity to perform the surgery. The ovarian artery was located and flow was inhibited with a 10 gauge suture tie. The ovary was completely removed while maintaining the integrity of the bursa and bursal cavity. The bioprosthetic ovary was inserted into the ovarian bursa, open side toward the oviducts and enclosed within the bursa by one or two stitches with 10 gauge nylon sutures. This was repeated for both ovaries. Sham surgeries were performed like these surgeries but with scaffolds that did not contain any cells. Seven mice with bioprosthetic ovaries and two mice with sham surgeries were mated with CD1 (white coat) males that had previously sired pups. Each female was paired with one male 4 days after surgery and housed together for 25 days or more. The presence of a solid plug within the mated female vagina indicated a successful mating. No additional mating was attempted. Pups resulting from this mating were identified as created from an egg released from the scaffold if the pup was *GFP+* or had a black coat colour. Pups that were *GFP−* with a white coat colour could not be determined as coming from the implant. The matings were performed in this way because these *GFP+* pups are homozygous lethal and, therefore, the *GFP+* follicles within the bioprosthetic only carry one *GFP+* copy. Therefore, we expect approximately half of the resulting pups from the bioprosthetic eggs to be *GFP+*.

### Statistical analysis

All data sets were analysed using GraphPad Prism software and expressed as the average±s.e.m. Statistical significance performed using one-way ANOVA with a Holm–Sidak's multiple comparisons test with *α*=0.05.

### Data availability

The authors declare that all data supporting the findings of this study are available within the paper and its Supplementary Information files.

## Additional information

**How to cite this article:** Laronda, M. M. *et al*. A bioprosthetic ovary created using 3D printed microporous scaffolds restores ovarian function in sterilized mice. *Nat. Commun.* **8,** 15261 doi: 10.1038/ncomms15261 (2017).

**Publisher's note**: Springer Nature remains neutral with regard to jurisdictional claims in published maps and institutional affiliations.

## Supplementary Material

Supplementary Information Supplementary Figures.

## Figures and Tables

**Table 1:** Description of three experimental designs used to investigate ovarian follicles in the 3D printed scaffolds.

**Experiment**	**Follicle size**	**Scaffold size, angle**	**Duration (mice per group)**
(a) *In vitro:* to test effects of architecture on survival to stimulate *in vitro* maturation	3–4 150–180 μm follicles per scaffold to visualize health of each under light microscopy	3–2 mm30°, 60°, 90°	2–8 days
(b) *In vivo:* to test vascularization and hormone restoration	Day 1: primordial, primary and small secondary follicles; Day 2: filled in with more follicles ≤180 μm; 40–50 follicles total	2 mm (to fit in bursa)60°	1 or 3 weeks post-surgery(16 OVX+implant; 5 OVX)
(c) *In vivo:* to test restorative organ function—mating, ovulation, live birth, lactation	Day 1: primordial, primary and small secondary follicles; Day 2: filled in with more follicles ≤180 μm; 40–50 follicles total	2 mm (to fit in bursa)60°	8–10 weeks post-surgery(7 OVX+implant; 2 OVX)

Ovariectomized animals with ovarian bioprosthesis implant are labelled as ‘OVX+implant'. Ovariectomized animals with a sham implant (3DP scaffold without cells) are labelled as ‘OVX'.
