# Supplementary material for: A bioprosthetic ovary created using 3D printed microporous scaffolds restores ovarian function in sterilized mice
Source: Nat Commun. 2017 May 16;8:15261. doi: 10.1038/ncomms15261 (PMC5440811; doi:10.1038/ncomms15261)
Supplement: Supplementary Information — Supplementary Figures. [file ncomms15261-s1.pdf]

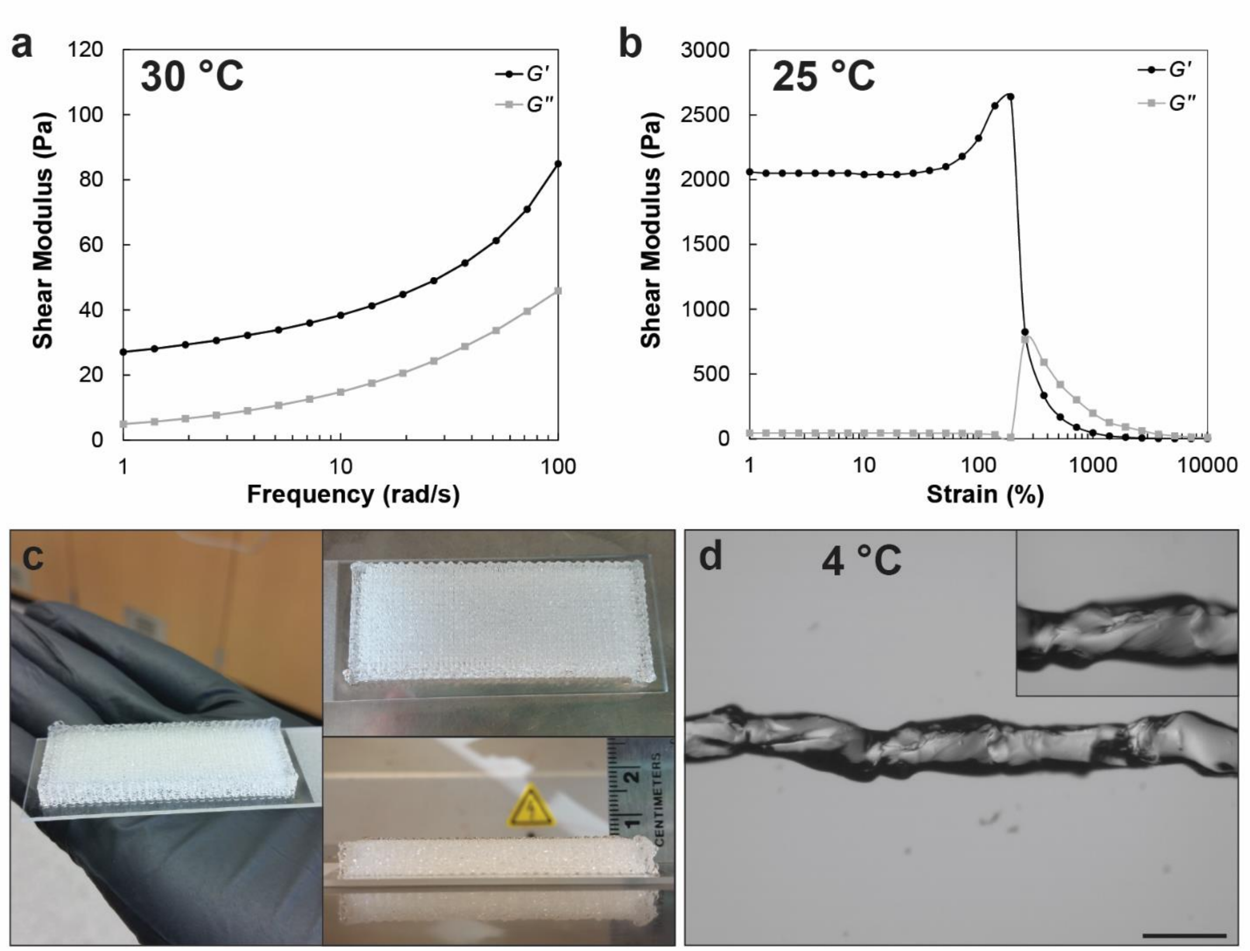

Supplementary Figure 1. Partially cross-linked gelatin is an ideal 3D biomaterial ink. a) Frequency sweep of 30 °C gelatin ink. b) Strain sweep of non-ideal, too robust 25 °C gelatin. The gel catastrophically failed at an earlier strain than 30 °C. c) 30 °C gelatin ink allows for printing of large, self-supporting objects on the scale of human tissues. Object shown 2 cm W x 5 cm L x 0.5 cm H = 5 cm^3. d) Fully cross-linked gelatin extruded into clumpy, inhomogeneous filaments while 30 °C were smooth and homogeneous. 10% gelatin at 4 °C (fully cross-linked) extruded from a 300 µm diameter nozzle at 2 bar pressure. Scale bar: 500 µm. Inset: magnified view of gel strand. Unlike partially cross-linked gelatin 30 °C, extrusion was very slow and was not continuous. Resulting strands were clumpy and roughly textured. Also unlike 30 °C gelatin, fully cross-linked gelatin was not able to be extruded from a 100 µm nozzle (up to a maximum pressure of 6 bar). Extrusion from a 200 µm nozzle was considered too slow for practical 3D printing.

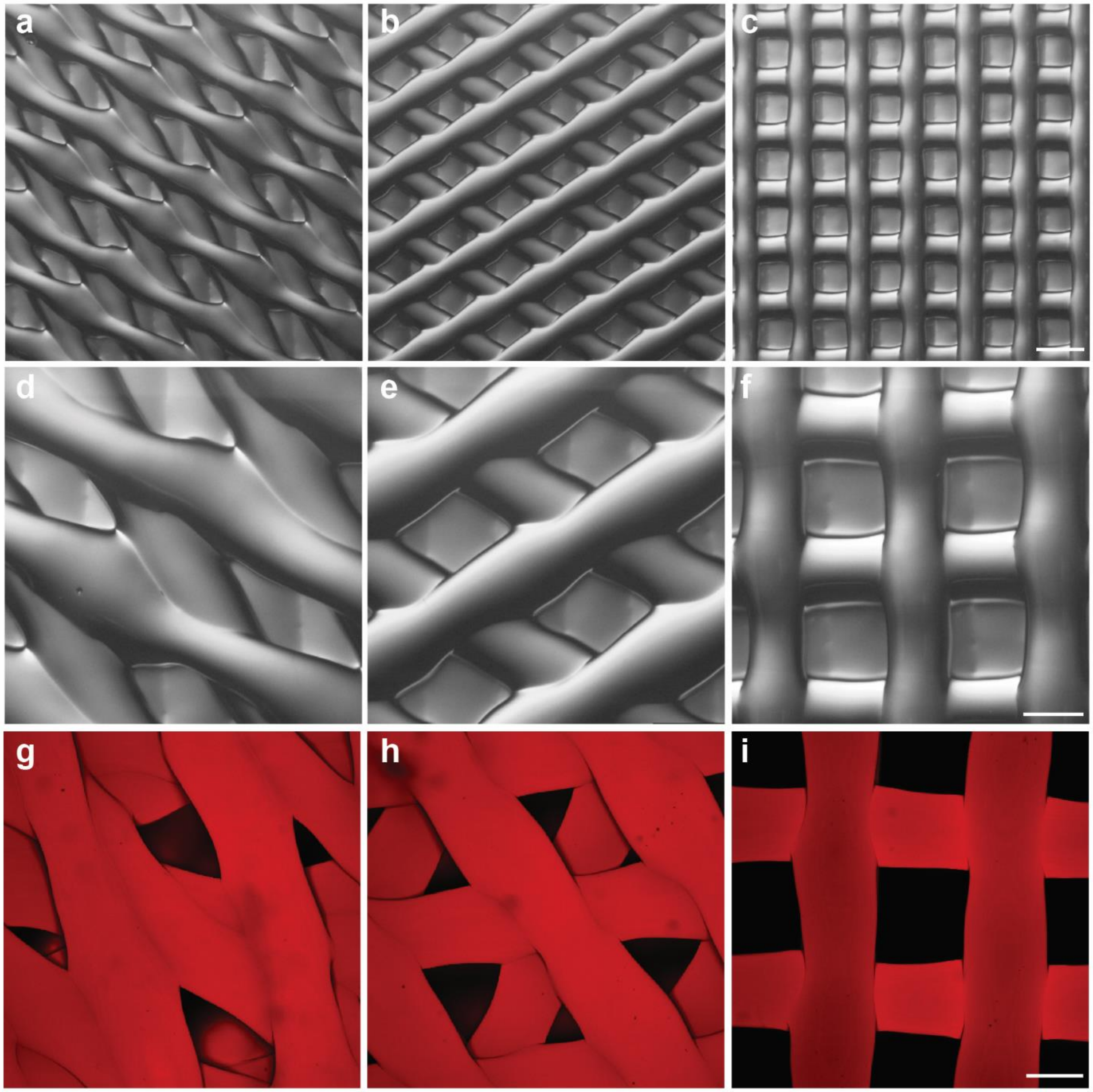

Supplementary Figure 2. Gelatin scaffolds printed with varying pore geometries. a, d, g,) 30° advancing angle. b, e, h) 60° advancing angle. c, f, i) 90° advancing angle. a-f) Light microscopy images. g-i) Confocal fluorescence microscopy images of rhodamine-labeled scaffolds. Maximum intensity projections of image stacks. Scale bars: a-c 500 µm; d-f 250 µm; g-i 200 µm.

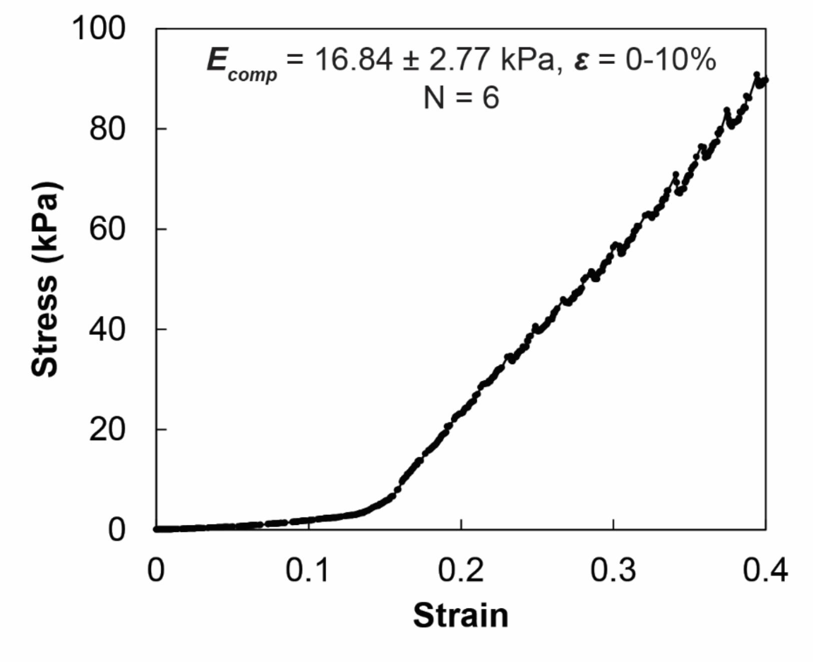

Supplementary Figure 3. Mechanical properties of cross-linked gelatin. Stress-strain curve from compression testing. 10% gelatin solution was cast into cylinders and cross-linked with EDC/NHS for 1 hour. The samples were in PBS for at least 24 hours. Prior to mechanical testing, gels were warmed to 37 °C. Compression testing was performed at 0.5 mm/min. Modulus was taken over 0–10% strain. Mean ± standard deviation.

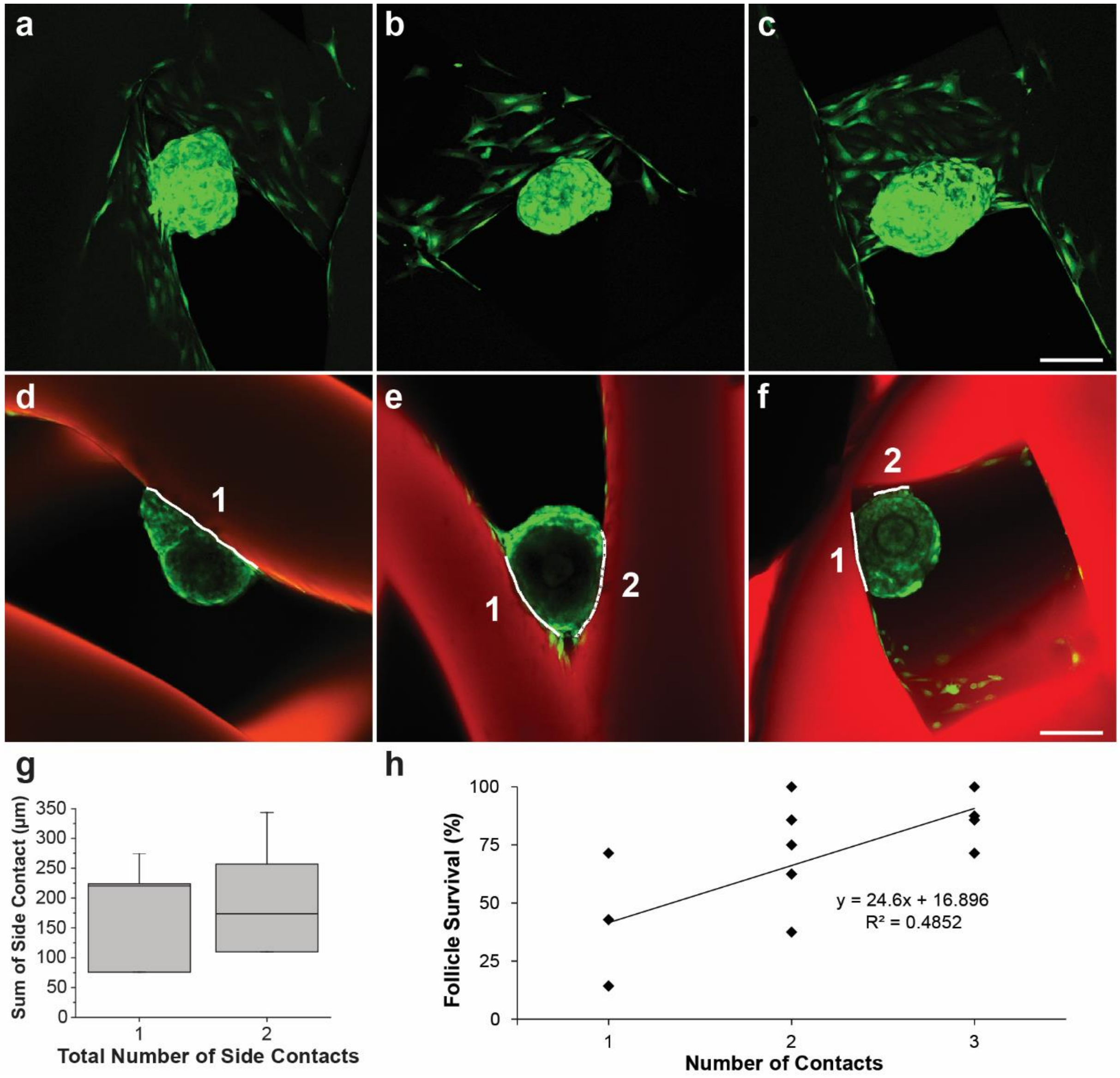

Supplementary Figure 4. Three-dimensional analysis of follicle-strut interactions. GFP^+ follicles were seeded into rhodamine-labeled scaffolds and analyzed by confocal fluorescence microcopy. a-c) Maximum intensity projections confocal image stacks. Only green channel is shown from images Fig. 2g-i. Stromal cells adhere to matrix and mediate follicle-strut interaction. d-f) Image slice that designates follicle-strut contact with the longest length in follicles making 1 (d) and 2 contact (e,f). Lengths: d_1 224.07 µm, e_1 158.84 µm, e_2 184.61 µm, f_1 129.62 µm, and f_2 58.98 µm. g) Side contact lengths summed. No significant difference between 1 and 2 side contact, $p = 0.59$. h) Correlation analysis of survival versus number of strut contacts. Linear regression shown. Pearson's $r = 0.6966$; p-value = 0.0027; $R^2 = 0.4852$. Scale bars: a-f 100 µm.

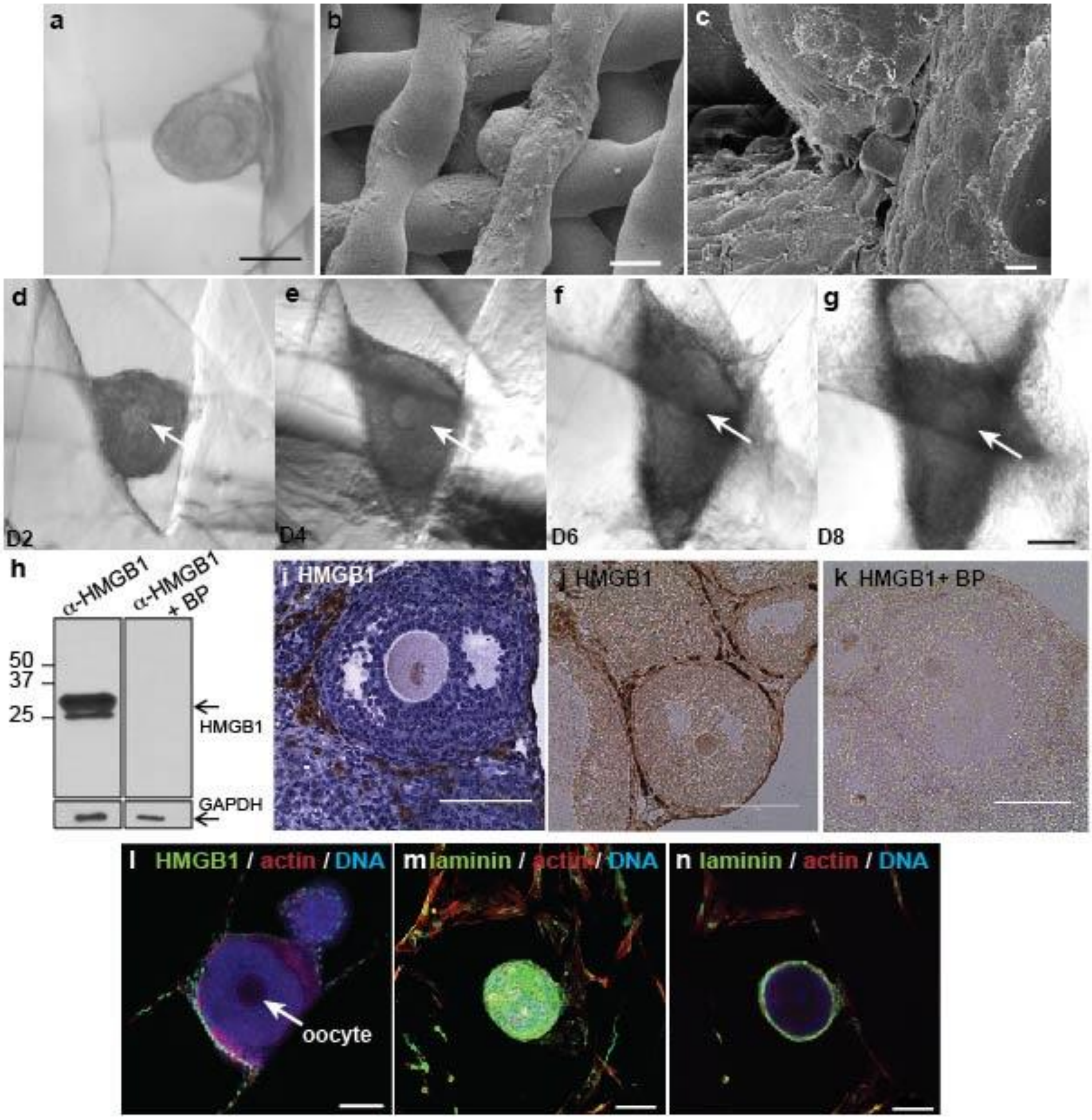

Supplementary Figure 5. Follicles become more integrated in scaffold pores over the 8 day culture period. a) Light microscopy images of follicle seeded in 60° advancing angle scaffold, adhered to a single strut on day 2 of culture. b-c) Scanning electron micrograph of same follicle after 6 days of culture with cells spread along scaffold struts. d-g) Light microscopy images of follicle seeded in 60° advancing angle scaffold pore corner. HMGB1 is expressed in stroma cells in the ovary. h) Western blot analysis identified HMGB1 protein expression in mouse ovarian lysate using an HMGB1 antibody. Pre-incubation of the HMBG1 antibody with a specific blocking peptide abrogated the signal (+ BP). i-j) HMGB1 localizes to stroma cells surrounding follicles and to oocyte nuclei (brown, hematoxylin counterstain in (i), blue). k) This detection is inhibited with pre-incubation of the HMGB1 antibody with blocking peptide (+ BP). l) Confocal fluorescence image slice of follicle at day 2 of cultured for 2 days in 60° scaffold. Immunostaining for HMGB1 (green), counter-stained for actin (red) and DNA (blue). Oocyte is visible within the center of follicle (white arrow). m-n) Maximum intensity projection of confocal fluorescence image stacks of follicle cultured 6 days in 60° scaffold and immunostained with laminin (green), actin (red) and DNA (blue). Scale bars: a, b, d-j, i, l-m, 100 μm; c, 10 μm; j-k, 200 μm.

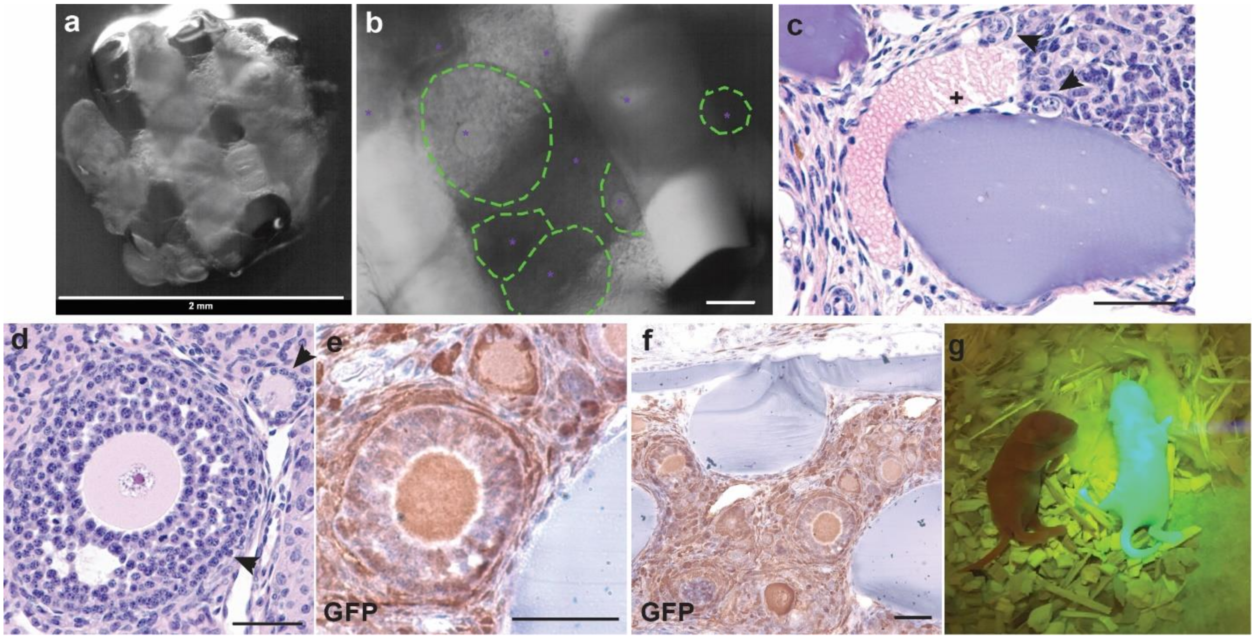

Supplementary Figure 6. Bioprosthetic ovaries support folliculogenesis, vascular infiltration, release of peptide hormones and live birth. a) Light microscopy image of bioprosthetic ovary composed of 2 mm 60° 3D printed scaffold and isolated murine follicles. b) Light microscopy image of bioprosthetic ovary with visible follicle boarders (outlined in green dotted line) and oocytes (purple *). c) H&E image of bioprosthetic ovary removed 3 weeks post-surgery containing large vessel (+) along strut and primordial follicles (arrows). d) H&E image of bioprosthetic ovary removed 8 weeks post-surgery containing a small primordial follicle next to a large pre-antral follicle. e-f) Immunohistochemical stain of GFP+ (brown) cells within the scaffolds struts (blue) of the implanted bioprosthetic ovary. g) GFP+ pup born from implant recipient next to GFP- pup from CD1 control colony. Scale bars: a 2 mm; b 100 μm; c-f 50 μm.
